# Suppurative thyroiditis caused by ingested fish bone in the thyroid gland: a case report on its diagnostics and surgical therapy

**DOI:** 10.1186/s12893-022-01542-x

**Published:** 2022-03-09

**Authors:** Anne Hendricks, Michael Meir, Mohammed Hankir, Christina Lenschow, Christoph-Thomas Germer, Michael Schneider, Armin Wiegering, Nicolas Schlegel

**Affiliations:** 1 Department of General, Visceral, Transplant, Vascular and Pediatric Surgery, University Hospital Wuerzburg, Oberduerrbacherstrasse 6, 97080 Wuerzburg, Germany; 2 Institute of Pathology, University of Wuerzburg, Josef-Schneider-Str. 2, 97080 Wuerzburg, Germany

**Keywords:** Fish bone, Foreign body ingestion, Thyroid gland, Thyroiditis, Case report, Surgical management

## Abstract

**Background:**

Accidental ingestion of fish bone is a common cause of otolaryngological emergency. Migration of the ingested bone into the thyroid gland, however, occurs very rarely. The associated clinical presentation, symptoms and duration of discomfort are also highly variable between patients and can be diagnostically challenging.

**Case presentation:**

Here, we report the case of a 71-year-old female patient presenting with an ingested fish bone that migrated into the right thyroid lobe as a rare cause of suppurative thyroiditis with the clinical features of sepsis. We outline the diagnostic approach, peri- and intraoperative management as well as complications. It is proposed that besides endoscopy, imaging methods such as ultrasound or computed tomography may be necessary to verify the diagnosis and location of an ingested fish bone. Prompt surgical removal of the foreign body and resection of the infectious focus is recommended to minimize the risk of local inflammation, recurrent nerve lesions and septic complications arising from the spread of infection.

**Conclusion:**

Fish bone migration into the thyroid gland is an extremely rare event, the successful detection and surgical management of which can be achieved through a careful interdisciplinary approach.

## Background

Foreign body ingestion, especially of fish bone, is a common otolaryngological emergency presenting at an ear-nose-throat (ENT) department. The associated symptoms typically include immediate pain in the pharynx or esophagus, foreign body sensation and severe discomfort. However, symptoms can be unspecific, and the identification of the foreign body can be diagnostically challenging. Due to its sharpness and firmness, a swallowed fish bone can cause injuries of the mucosa, penetrate the pharynx, esophageal and gastric wall or even perforate the intestine. Depending on the location, this may result in various complications such as local infections, abscess formation, peritonitis, sepsis, nerve or vascular injury [1–3]. Strategies to remove the fish bone and therapeutic management of complications varies from endoscopic intervention to surgical exploration [4–7].

Extraluminal migration of ingested fish bone is an extremely rare event and less than 10 cases of migration within the soft tissue of the neck have been described so far. Here, we report an unusual case of migration of a fish bone into the thyroid gland, causing purulent thyroiditis, local inflammation and the clinical features of sepsis. We present our approach and discuss the diagnostic procedures as well as peri- and intraoperative surgical management.

## Case presentation

A 71-year-old female patient presented at the ear, nose and throat (ENT) emergency department with progressive stabbing pain and foreign body sensation in the throat after having consumed a gilthead earlier the same day. Accidental ingestion of fish bone was suspected although this could not be confirmed from the clinical examination alone, which included inspection of the oral cavity and throat. The persistence of symptoms led to hospitalization of the patient, where endoscopy of the upper respiratory tract and upper gastrointestinal tract and blood tests were performed on consecutive days, but revealed no abnormalities. Since the patient’s symptoms eventually improved, she was discharged from hospital.

Four days later, the same patient returned to the ENT emergency department with a high fever (40.2 °C), general feelings of illness, progressively sore throat and odynophagia. Upon re-admission, blood tests revealed signs of ongoing inflammation with significantly increased C-reactive protein levels of 18.05 mg/dl (normal range ≤ 0.5 mg/dl) while leukocytes were in a high-normal range. Thyroid-stimulating-hormone (TSH) was slightly suppressed at 0.09mIU/l, (normal range of 0.3–4.0 mIU/l) while levels of T3 and T4 were fine.

During clinical examination of the neck, the patient indicated tenderness upon palpation of the right side and swollen lymph nodes were detected. Ultrasound did not identify any signs of abscess formation. However, the right thyroid lobe appeared inhomogeneous and was enlarged compared to the left thyroid lobe despite normal perfusion. No other abnormalities were identified during a comprehensive diagnostic workup of the lung, abdomen, ears, nose or throat. Concerning the patient´s history, no pathologies of the thyroid gland, throat, neck and esophagus were diagnosed previously and no intervention was ever performed in this anatomic region. Based on the soft tissue inflammation of the neck, an intravenous antibiotic treatment with clindamycin was initiated.

**Fig. 1:**
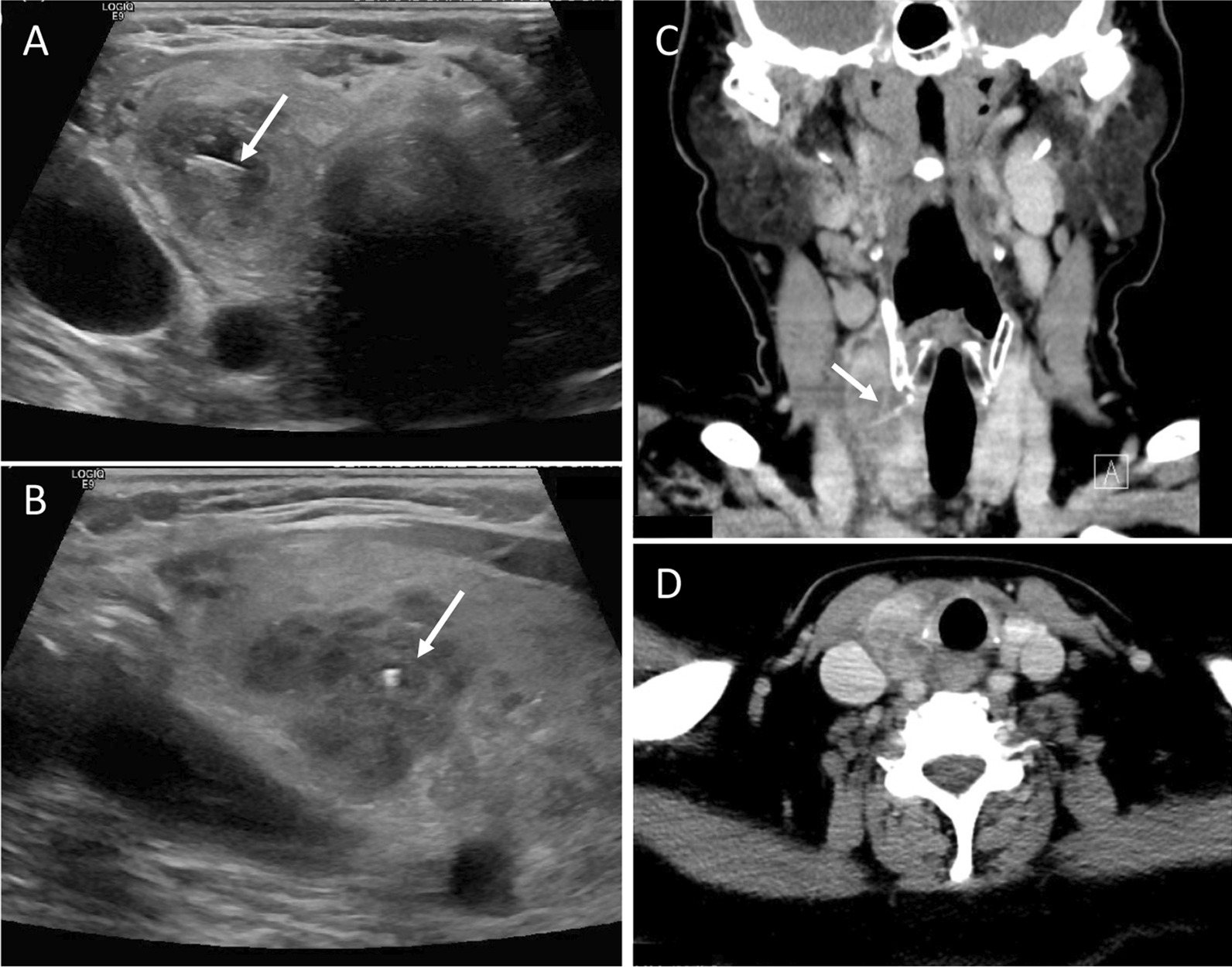
Diagnostic imaging of the neck. **A** and **B** CT scan of head and neck showing an inhomogeneous, swollen right thyroid lobe with a hypodense lesion (2.2 cm) as indicated by the arrows and inflammatory reaction of surrounding tissue. The left thyroid lobe appears unaltered with a normal size. No abscess was detected. **A** A thin hyperdense structure is present in the right thyroid lobe that was later identified to be a fish bone. **C** and **D** Ultrasound diagnostic confirmed the diagnosis of an enlarged thyroid lobe with a volume of 28.6 ml compared to 9.2 ml of the left thyroid lobe. A central, circumscribed area was identified in the right thyroid lobe, mainly hyperechoic with hypoechoic fractions and paranodular homogenous thyroid tissue without increase of perfusion. Within the inhomogeneous area, a sharp foreign body was identified. This was suspected to be a fish bone (arrows)

A CT scan of the neck was performed on the next day. This revealed a swollen right thyroid lobe with a 2.2 cm hypodense lesion lacking clear margins indicative of thyroiditis (Fig. 1A, B). Furthermore, the retrolaryngeal tissue adjacent to the esophagus was affected.

The patient was then referred to the department of nuclear medicine for further evaluation of the thyroid gland. Another ultrasound was carried out by a specialist for nuclear medicine who diagnosed acute thyroiditis. The right lobe had a central, circumscribed area of altered thyroid tissue (2.0 × 1.9 × 3.1 cm) with mainly hypoechoic fractions and an area of paranodular homogenous thyroid tissue without increased perfusion. Moreover, a spiky and sharp hyperechoic structure spanning from the level of the trachea to the lateral thyroid border was identified (Fig. 1C, D).

In view of these findings and considering that the patient consumed fish a week prior to the ultrasound scan, the migration of a fish bone into the right thyroid gland was suspected. Consequently, no continuative diagnostics like thyroid-scintigraphy were performed. The patient was then transferred to the surgical department and after interdisciplinary discussion, surgical exploration was indicated.

**Fig. 2:**
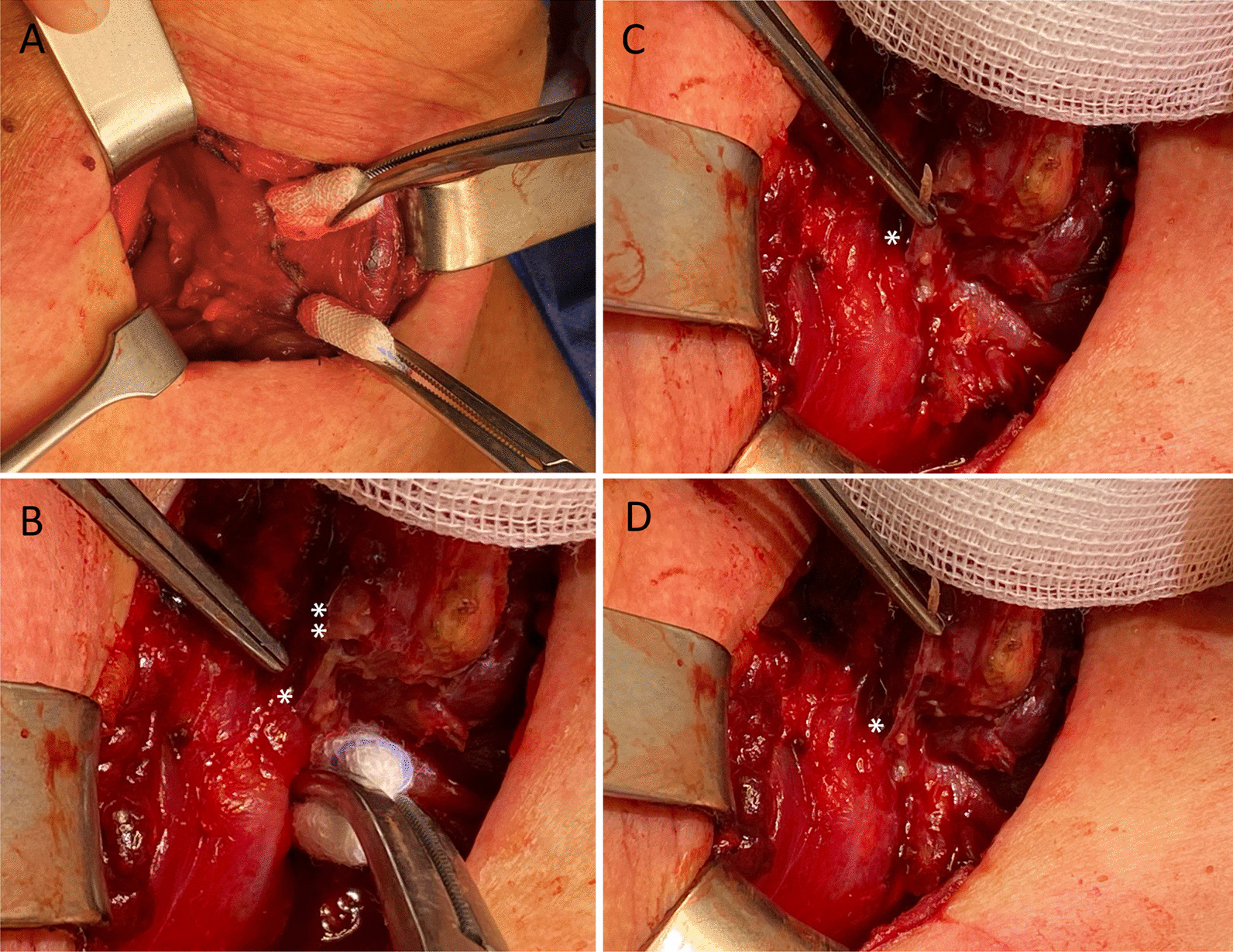
Intraoperative pictures during the surgical exploration. **A** Preparation of the parathyroidal space revealed local inflammation. The right thyroid lobe presented with suppurative tissue alterations and was adherent to esophageal wall and thyropharyngeal muscle. **B** The fish bone was identified perforating the esophageal wall (arrow*) and penetrations the right thyroid lobe dorsally (arrow **). **C**, **D** The fish bone was removed carefully

Intraoperatively, the right thyroid lobe was found to be severely inflamed with adhesions to surrounding tissue especially the thyropharyngeal muscles and esophagus. Careful preparation was thus necessary to prevent surgical trauma of the recurrent laryngeal nerve or esophagus. Finally, the fish bone was identified penetrating the right thyroid lobe dorsally while still perforating the esophageal muscularis/pharynx (Fig. 2). While the perforation zone was near the recurrent laryngeal nerve, it remained unscathed. The fish bone was removed gently (Fig. 2). Afterwards, hemithyroidectomy of the inflamed right thyroid lobe was performed. It was not possible to detect parathyroid glands within the inflamed tissue. Intraoperative neuromonitoring of the recurrent laryngeal and the vagal nerves revealed normal electromyographic signals before and after the thyroid lobe was removed indicative of intact recurrent laryngeal nerve function. Macroscopically, no lesion of the esophagus was visible and therefore, no suture was needed. After extensive rinsing of the wound cavity, a wound (redon) drain was placed on the surgical site. Surgery was successfully completed, and the patient was observed postoperatively.

**Fig. 3:**
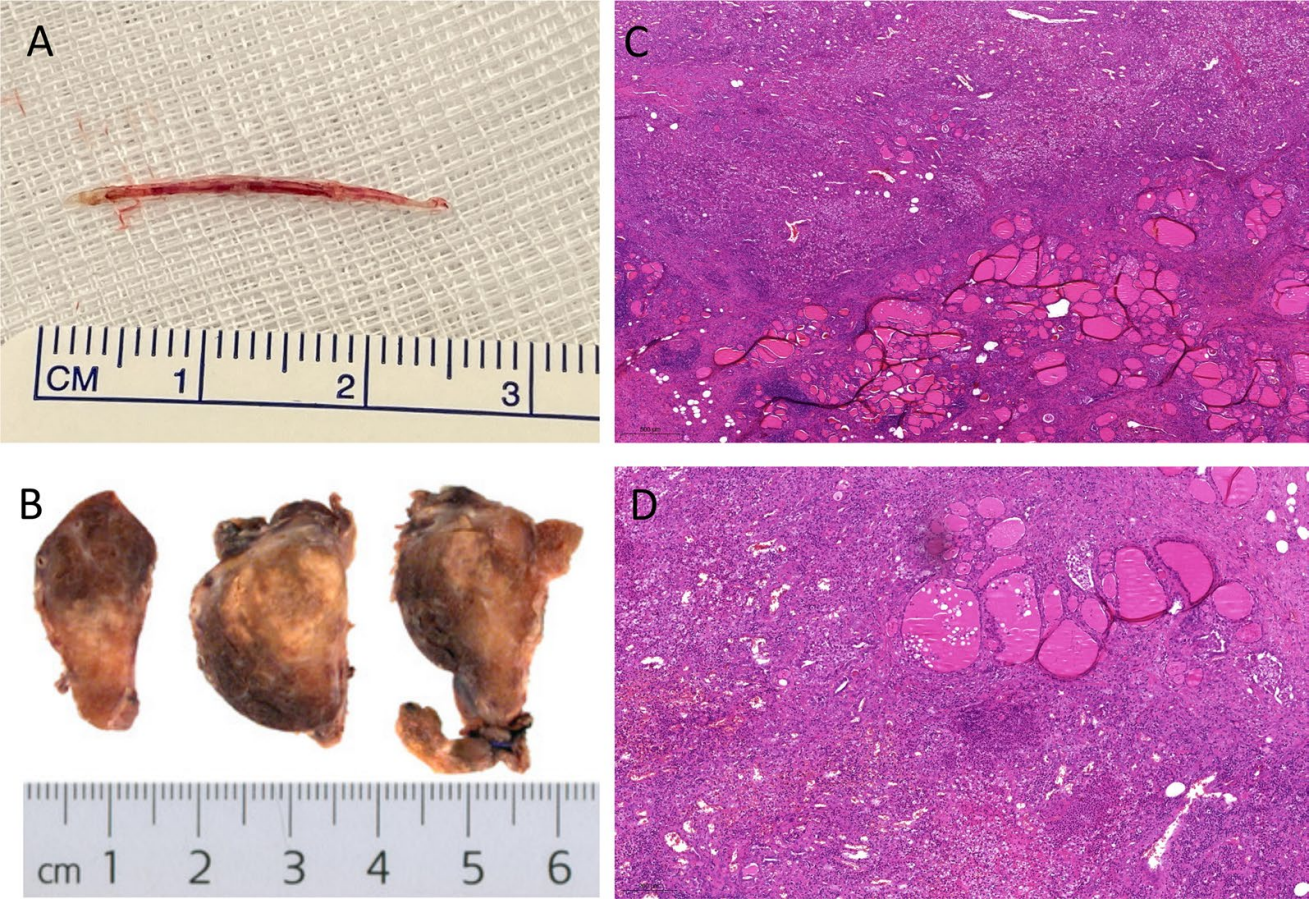
Histopathological workup. **A** The removed fish bone measures 2.6 cm. **B** Resected right thyroid lobe measuring 6.0 × 4.0 × 2.0 cm showing inflammatory altered tissue. **C**, **D** HE staining of histopathological sections of the right thyroid lobe. Representative images show histiocytic immune reaction with infiltration of segmented eosinophilic granulocytes as equivalent for a purulent infection of the thyroid gland with surrounding granulomatous and fibrous reaction

The removed fishbone measured 2.6 cm (Fig. 3A). The resected thyroid tissue was sent to the pathologist for further histopathological examination. This revealed purulent inflammation with a focal foreign body giant cell reaction and lymphofollicular, partly chronic resorptive, histiocytic thyroiditis (Fig. 3C, D). Additionally, nodular goiter was detected. Retrospectively, neither pre- nor intraoperatively any lesion or pathology was found which could explain the unusual localization of the fishbone.

Postoperatively, antibiotic treatment was terminated immediately. The patient reported an improvement of pain and recovered quickly. Vocal cord mobility was checked by an ENT specialist and was normal. Dysphagia was no longer reported and the patient was able to receive oral nutrition immediately. No fever occurred and blood tests revealed a normalization of inflammatory markers within 4 days and calcium levels at every timepoint. The drain was removed 3 days after surgery with no need for additional endoscopy. The patient was discharged in good condition and did not develop recurrent symptoms.

## Discussion

While ingestion of fish bone is an occasional accident requiring medical attention, the migration of fish bone into the thyroid gland as reported here is extremely rare. This can cause difficulties in diagnosis and uncertainty regarding the best treatment option.

When our patient presented at the ENT emergency department for the first time, clinical examination failed to detect a foreign body around the neck or reveal the reasons for her symptoms, although patient history indicated accidental ingestion of fish bone as a possible cause. Only upon the patient’s second admission to hospital when her condition deteriorated and more extensive diagnostics were performed, could the migrated fish bone be found in the right thyroid globe.

There are 18 cases of fish bone migration into the thyroid gland described in the literature (reviewed in [8]). A comparison of individual cases attests to how variable patient histories, time courses and clinical presentations can be. While an immediate onset of symptoms after consumption of fish facilitates a prompt diagnosis, unspecific symptoms for months or a resolution and return of pain over time can delay or even complicate diagnosis [8–10]. Consequently, the detection of a migrated fish bone might become an interdisciplinary diagnostic challenge.

The standard diagnostic workup mainly depends on the presentation of the patient. For patients with an acute onset of symptoms after ingestion of a sharp object, flexible endoscopy of the larynx, pharynx and upper esophagus is the usual approach after clinical examination [11]. It is noteworthy that a negative endoscopy does not prove the absence of a foreign body. This can commonly be explained by its movement to the stomach or intestine and for fish bones in particular, migration to extraluminal tissue like the soft tissue of the neck [12, 13]. Furthermore, the mucosa can present without any lesion or pathology even after migration of the fish bone [10]. Therefore, further diagnostics are recommended, especially when symptoms do not resolve or quickly worsen and when signs of infection develop.

The patient presented here first received a CT- scan when she was re-admitted with infectious symptoms. While this failed to clarify the problem, it pointed to the thyroid gland as a focus of the symptoms. Ultrasound of the neck was the next crucial step in detecting the fish bone in the thyroid gland. Notably, only upon the second ultrasound examination, performed by a specialist in nuclear medicine experienced in thyroid ultrasound, was the fishbone able to be detected. This underlines the impact of investigator experience in such cases. In addition, it can be assumed that local inflammation was more pronounced after 4d when the second ultrasound was performed which could have facilitated the diagnosis. Our case is in line with previous studies showing that CT and ultrasound are both appropriate and complementary imaging techniques to detect foreign bodies [2, 8, 10]. On the one hand, ultrasound is broadly available, cost effective and without ionizing radiation and can be used by an experienced investigator to detect foreign bodies, tissue alterations or liquid formations [14]. On the other hand, CT offers a detailed presentation of the foreign body, its position compared to surrounding tissue and potential local complications [1]. Additionally, if the cause of thyroiditis is not known, CT provides a more comprehensive reconstruction of the neck and can uncover pathologies of different compartments [15, 16].

The urgency of therapy highly depends on the etiology of thyroiditis and patient condition. In cases of foreign body migration causing suppurative thyroiditis, antibiotic treatment is immediately initiated in most cases but without delaying causal therapy and resection of the infectious focus. Otherwise, suppurative thyroiditis can progress to retrotracheal/retropharyngeal abscess formation, mediastinitis and sepsis to become life-threatening [17]: Interestingly, Cavelier et al. provided preoperative antibiotic treatment for 15 days before surgical treatment of the thyroid gland after fish bone migration suggesting delayed surgery is viable [10]. However, as in most cases of foreign body migration, early operative therapy is recommended to prevent long-term inflammation, tissue deterioration and scar formation which can complicate surgery.

The degree of surgical resection required to remove a foreign body in the thyroid gland can vary from none to hemithyroidectomy to complete thyroidectomy [2, 18]. Importantly, resection of infectious tissue should be ensured to prevent the need for repeat surgery, which comes with increased risk of complications including recurrent nerve injury. After complete removal of the foreign body and any infectious tissue, antibiotic treatment can be quickly discontinued.

Surgical exploration of the thyroid gland during acute suppurative inflammation is challenging as the inflammatory process causes adverse tissue reactions. Increased sensitivity and perfusion of the tissue, adhesions of surrounding tissue as well as thyroid gland and esophagus are common. Therefore, careful preparation is necessary to prevent lesion of the recurrent laryngeal nerve and injury of the upper esophagus as this would cause severe long-term consequences for the patient. Preservation of the parathyroid glands is ideal even if their identification is hindered by surrounding inflamed tissue. As described here, another study reported no need to suture the esophageal wall after fish bone removal from the thyroid gland [8]. However, Ohbuchi et al. described a case of fish bone migration into the thyroid gland with cutaneous fistula and contact of the fish bone to the esophageal wall which required suturing [2].

Besides intraoperative complications, leakage of the esophagus could become a postoperative complication necessitating endoscopy. In our case, no lesion of the mucosa was seen during preoperative endoscopy and the esophageal wall was intact during surgery. Therefore, we did not regard postoperative endoscopy as necessary. In cases with broader inclusion of the esophagus to the infectious side, visible lesions or inadequate improvement of the patients´ condition, postoperative endoscopy might be helpful for early detection of complications and intervention. If a persisting esophageal lesion is seen, newer interventional therapeutic approaches like endoscopic vacuum-assisted closure applied to esophageal perforation or leakage can be a helpful tool to avoid re-surgery or progression of the defect [19].

For most reported cases, no postoperative complications were observed, oral food intake was possible the next day and patients recovered quickly. Since the fish bone only caused a minor lesion to the esophagus future scarring should not be a problem. Therefore, we expect no long-term complications and no increased risk for swallowing of foreign bodies or for a recurrent fish bone migration. Furthermore, there is no evidence in the literature for potential long-term problems.

The main limitation of this case report is that migration of fish bone into the thyroid gland is extremely rare and therefore may be of low interest. On the other hand, only few surgeons have experiences to recognize this problem and there is little knowledge on the appropriate therapeutic steps. Therefore, one strength of this case report is that it provides a detailed description of diagnostics and therapy including the discussion of a high number of potential variables.

In conclusion, fish bone migration into the thyroid gland is an extremely rare event, the successful detection and treatment of which can be achieved with careful interdisciplinary cooperation. The diagnostic approach should be adapted to the patients’ presentation. Ultrasound of the neck can facilitate the diagnosis. In cases of acute infection, immediate therapy is required. Surgical removal of the fish bone and inflamed tissue is the therapy of choice. Inflammation can cause adhesions, tissue alterations and inclusion of the esophagus to the local process. Surgery should therefore be conducted by an experienced endocrine surgeon.

## Data Availability

Data sharing is not applicable to this article as no datasets were generated or analyzed during the current study.

## Abbreviations

ENT: Ear-nose-throat
TSH: Thyroid-stimulating-hormone
CT: Computer-tomography
HE: Hematoxylin and eosin
